# Basidiobolomycosis of the Colon Masquerading as Stenotic Colon Cancer

**DOI:** 10.1155/2011/685460

**Published:** 2011-10-16

**Authors:** M. Ezzedien Rabie, Ismail El Hakeem, Mubarak Al-Shraim, M. Saad Al Skini, Salim Jamil

**Affiliations:** ^1^Department of Surgery, Armed Forces Hospital Southern Region, P.O. Box 101, Khamis Mushait, Saudi Arabia; ^2^Department of Pathology, College of Medicine, King Khalid University, Abha, Saudi Arabia

## Abstract

*Basidiobolus ranarum* is a widespread saprophyte fungus with pathogenic potential. It affects mainly the subcutaneous tissues of the trunk and limbs. Relatively recently, occasional reports of gastrointestinal basidiobolomycosis appeared in the literature. Due to the rarity of the condition and the nonspecific presenting features, the correct diagnosis is usually hard to reach. In this paper, we describe the clinical course of an otherwise healthy female, who presented with a colonic mass. She received subtotal colectomy followed by oral itraconazole, with successful outcome.

## 1. Introduction: Mucorales

The class Zygomycetes includes two fungal orders: Mucorales and Entomophthorales, with extremely different pathogenic potentials. Mucorales affect only the immunocompromised patient causing mortality in excess of 60% in those affected while entomophthorales, which include Basidiobolus and Conidiobolus genera, affect the immune competent individual, causing principally chronic infection of the subcutaneous tissue [1].

The fungus *Basidiobolus ranarum * is present in soil, decaying vegetable matter, and the intestines of amphibians, reptiles, fish, and insectivorous bats. The infection is presumably acquired through insect bites or exposure to the fungus following minor trauma to the skin [2].

## 2. Case Report

A 25-year-old female presented with colicky epigastric pain and nausea for three months, associated in the last month with weight loss and rectal bleeding.

On examination, she looked relatively well; her blood pressure was 104/60 mmHg, pulse 68/minute, respiration rate 20/minute, and temperature 37°C. Abdominal examination revealed a 5 × 8 cm firm, ill-defined mass in the left side of the epigastrium.

Her laboratory works showed leucocytosis (12.8 × 10^9^/*μ*L, reference range 4–11 × 10^9^) and apart from low albumin (21 gm/dL, reference range 34–48), her liver functions, renal values, and electrolytes were normal.

**Figure 1:**
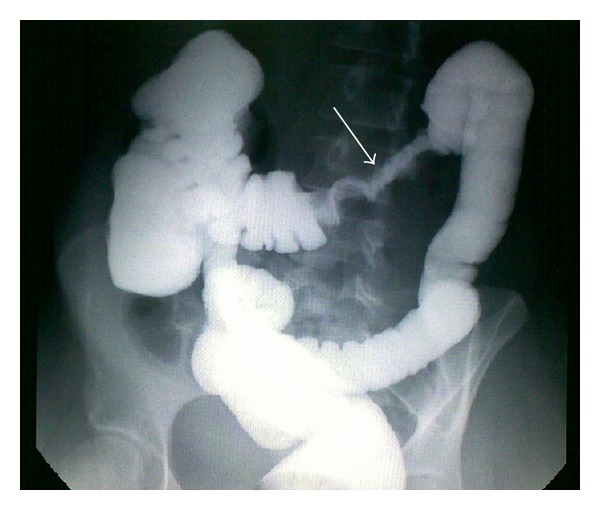
Barium enema showing apple core appearance and shouldering (white arrow), suggestive of colon cancer.

Ultrasound/computerized axial tomography scan showed a large mass in the left half of the transverse colon with mural thickening and irregular narrowing of the lumen. Multiple enlarged mesenteric lymph nodes were also seen. Barium enema showed a long irregular stricture of the distal transverse colon (Figure 1).

Colonoscopy revealed an incomplete stricture of the middle and distal transverse colon with mucosal ulceration. Biopsies were taken and histopathology showed chronic active colitis with extensive ulcerations.

**Figure 2:**
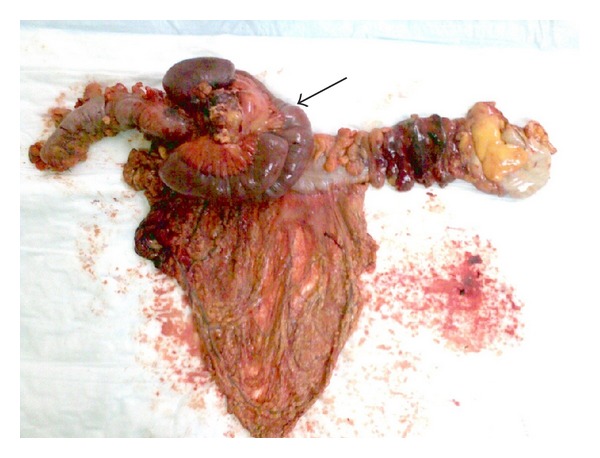
Resected colon with adherent small bowel loop (black arrow).

As the clinical picture was so much suggestive of colon cancer, the patient was subjected to laparotomy where a firm mass was found in the left half of the transverse colon, to which a jejunal loop was adherent. Multiple enlarged lymph nodes were also found in the root of the mesentery. Subtotal colectomy with excision of the adherent 45 cm jejunal loop was performed and bowel continuity restored with ileosigmoid colostomy (Figure 2).

**Figure 3:**
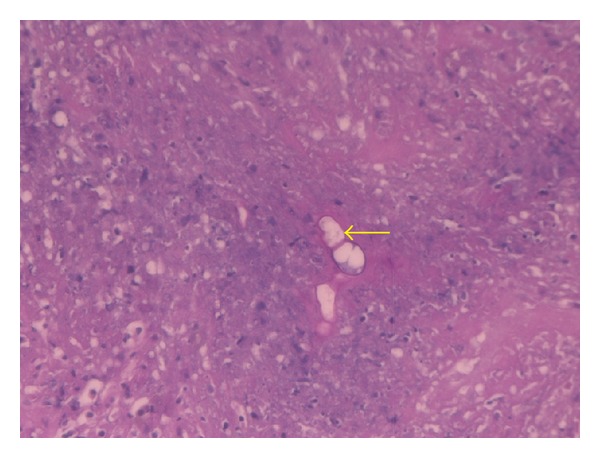
Periodic acid-Schiff stain showing fungal forms (yellow arrow) surrounded by Splendor-Hoeppli phenomenon in a background of acute suppurative inflammation and necrosis. (PASX40).

Histopathology of the resected specimen showed granulomatous reaction with areas of necrosis and acute suppurative inflammation. Broad, thin-walled, occasionally septated hyphae, surrounded by dense eosinophilic reaction (Splendore-Hoeppli phenomenon) were seen, which suggested the diagnosis of basidiobolomycosis (Figure 3). Oral itraconazole, in a daily dose of 200 mg, was started. In her follow-up outpatient visits, the patient remained well, her investigations, including liver and renal function tests, were normal, and she is still under itraconazole treatment.

## 3. Discussion

Basidiobolomycosis is a rare infection caused by the fungus *Basidiobolus ranarum*, of the order Entomophthorales, of the class Zygomycetes [3]. Our MEDLINE search yielded 27 published cases with the largest series involving only seven cases from Arizona, USA [4].

The infection, encountered chiefly in East and West Africa, Indonesia, and India, usually affects the trunk and limbs, producing chronic subcutaneous infection [1]. Other rare sites include the gastrointestinal tract [1, 3, 4], which proved fatal in exceptional occasions [5] and the nasal sinuses [6]. 

How the fungus reaches the gastrointestinal tract is not clear, but ingestion of soil or animal faeces or food contaminated by either, seems to be the possible route [4].

The disease, which has been described relatively recently, has radiological features suggestive of inflammatory bowel disease or malignancy. However, contrary to these pathological entities, leucocytosis and eosinophilia are usually present [4]. In the case presented here, our patient had leukocytosis with no eosinophilia, and as previously reported [4], leukocytosis resolved after surgical resection.

Preoperatively, we diagnosed the case as a stenotic malignant lesion, or, though appeared less likely, inflammatory bowel disease, which is a common pitfall in such condition [4, 7].

The difficulty in diagnosing the condition is multifactorial. Firstly, the clinical presentation is non specific. Secondly, there is no identifiable risk factors [4]. Thirdly, as the causative agent lies deep beneath the mucosa, colonoscopic biopsies may be nonrepresentative. More often than not, the biopsy reveals non specific inflammation or granulomatous reaction. In exceptional cases, fungal hyphae may be detected. However, in suspected cases, tissue culture for fungi may provide the clue for the correct diagnosis [8]. The fourth confounding factor and contrary to expectations, victims of this infection are usually healthy immunocompetent subjects [8], in contrast to the commoner opportunistic fungal infections.

As the condition is rarely, if ever, diagnosed preoperatively, the diseased segment is usually excised [8], with impunity, as surgical resection, together with prolonged administration of azoles, are the main pillars of treatment [4].

The correct diagnosis is usually unveiled by the histopathological examination of the resected specimen or, by isolation of *Basidiobolus ranarum* from the cultured tissues [4]. The typical morphology of the fungus has been previously described. Histologically, there are irregularly branching thin-walled hyphae, occasionally septated and surrounded by a thick eosinophilic cuff (Splendore-Hoeppli phenomenon) [4, 8–11]. Moreover, the involved bowel segment shows marked mural thickening, fibrosis, prominent eosinophilic infiltration, and granulomatous reaction. In the case presented here, these features were present and enabled us to discover the true nature of the pathology. However, the irrefutable diagnosis needs tissue culture [8], which, due to lack of suspicion, was not carried out in our case. In the absence of culture, serodiagnosis is a useful confirmatory adjunct which could also be used to follow the response to treatment [4].

In vitro studies and clinical reports showed the effectiveness of fluconazole, itraconazole, ketoconazole, and miconazole against Basidiobolus species, [2, 12], and prolonged treatment for up to two years is usually needed [4, 8, 13]. Reports about the effectiveness of potassium iodide are conflicting [14, 15]. Follow up of the response to treatment is generally done by serial CT scan to detect regression or otherwise of the “pseudotumours” and by serological tests to detect the disappearance of specific antibodies [4].

In the case presented here, the diagnosis was unsuspected till the histopathologic examination of the resected colonic mass showed resemblance to the published features of basidiobolomycosis. Itraconazole therapy is planned to continue till the radiologic/serologic tests demonstrate complete eradication of the infection.

## 4. Conclusion

Gastrointestinal basidiobolomycosis is a recently recognized disease, the clinical features of which resemble those of inflammatory or neoplastic bowel disease. Whenever these diagnoses are entertained, basidiobolomycosis might be considered in the differential diagnosis.
